# Emerging therapies for relapsed/refractory multiple myeloma: CAR-T and beyond

**DOI:** 10.1186/s13045-021-01109-y

**Published:** 2021-07-23

**Authors:** Christopher T. Su, J. Christine Ye

**Affiliations:** 1 Department of Internal Medicine, University of Michigan, Ann Arbor, MI 48109 USA; 2 Rogel Cancer Center, University of Michigan, Ann Arbor, MI 48109 USA

**Keywords:** Multiple myeloma, Review, First in human trials, Chimeric antigen receptor, CAR-T, Bispecific antibody, T cell engager, Antibody–drug conjugate, Monoclonal antibody, Small molecule inhibitor, Targeted therapy

## Abstract

The pace of innovation of multiple myeloma therapy in recent years is remarkable with the advent of monoclonal antibodies and the approval of novel agents with new mechanisms of action. Emerging therapies are on the horizon for clinical approval with significant implications in extending patient survival and advancing closer to the goal of a cure, especially in areas of immunotherapy such as chimeric antigen receptor T cells, bispecific T cell engager antibodies, antibody drug conjugates, newer generations of monoclonal antibodies, and small molecule inhibitor and modulators. This review provides an update of current myeloma therapeutics in active preclinical and early clinical development and discusses the mechanism of action of several classes of novel therapeutics.

## Introduction

Although multiple myeloma remains an incurable disease, the 5-year relative survival rate has nearly doubled in the last 20 years, from 32.1% in 1996 to 54.9% in 2016 based on National Cancer Institute statistics. Numerous treatment options have entered the myeloma therapeutic landscape in the last 5–10 years, significantly extending progression-free survival (PFS) and overall survival (OS). In addition to IMIDs (immunomodulatory agents) and PIs (proteasome inhibitors), daratumumab, an anti-CD38 antibody initially approved for relapsed/refractory patients, is now moving into the frontline setting for newly diagnosed multiple myeloma, therefore adding a third gold standard in the newly diagnosed multiple myeloma therapeutic paradigm [1, 2].

**Fig. 1:**
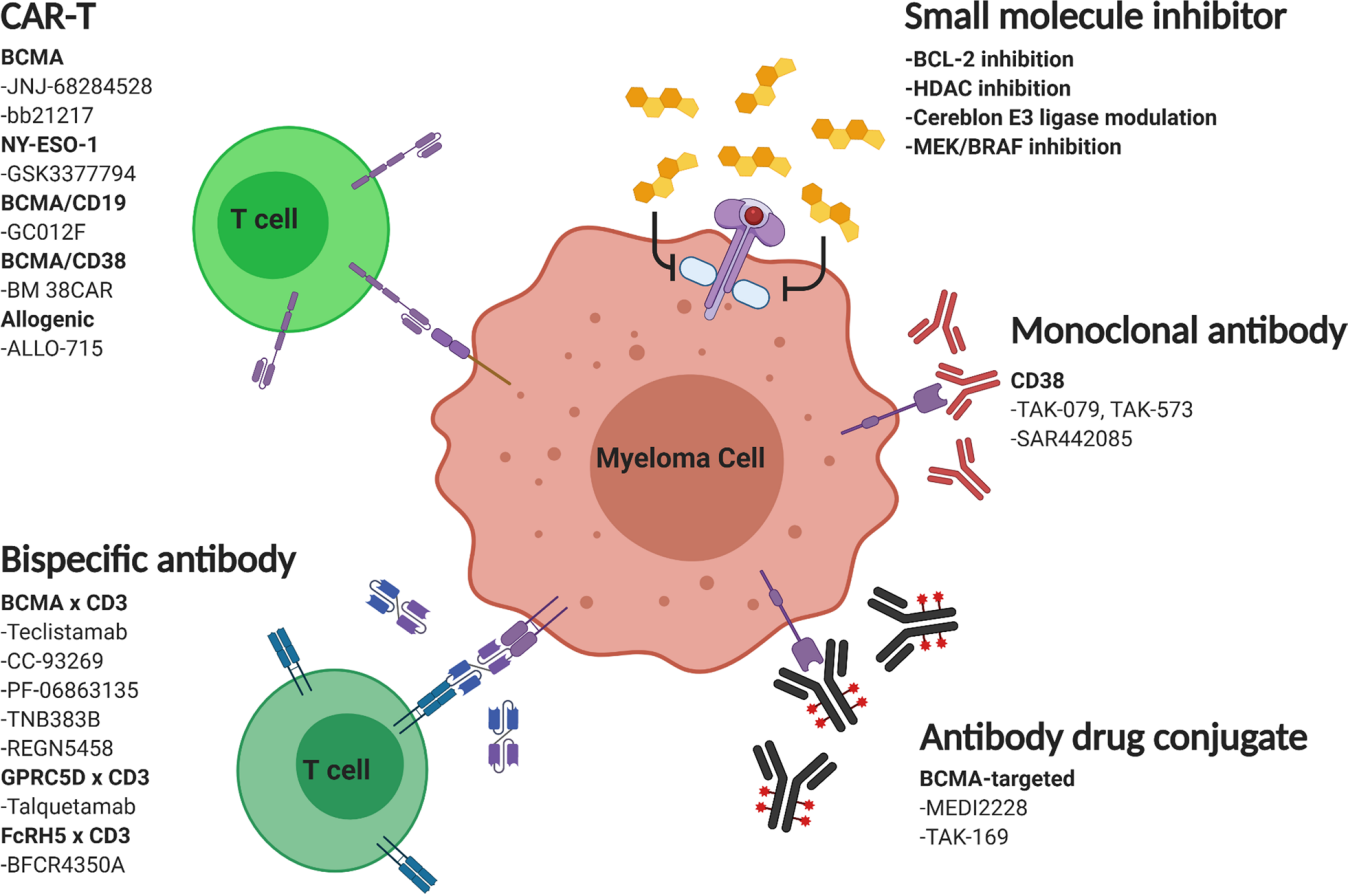
Mechanisms of action of emerging multiple myeloma therapies

Relapsed/refractory multiple myeloma (RRMM) is an extremely active area of research, largely due to the nature of myeloma disease heterogeneity and clonal evolution throughout the disease progression [3, 4]. Drug approvals in the last couple of years include selinexor (XOP1 nuclear export inhibitor) and isatuximab (another anti-CD38 monoclonal antibody), which were approved by the Food and Drug Administration (FDA) in July 2019 and March 2020, respectively. Most recently, attesting to the vibrancy of ongoing myeloma research, the first-in-class B-cell maturation antigen (BCMA)-targeted antibody–drug conjugate (belantamab mafodotin) was approved in August 2020 [5], and BCMA-targeted CAR-T cell therapy (idecabtagene vicleucel) in March 2021 [6]. Currently, the landscape of emerging multiple myeloma clinical trials is diverse and encouraging, including chimeric antigen receptor (CAR)-T cells, bispecific T-cell engager antibodies (BiTEs), antibody–drug conjugates (ADCs), newer generations of monoclonal antibodies (MoAbs) and small molecule inhibitors/modulators (Fig. 1).

**Fig. 2:**
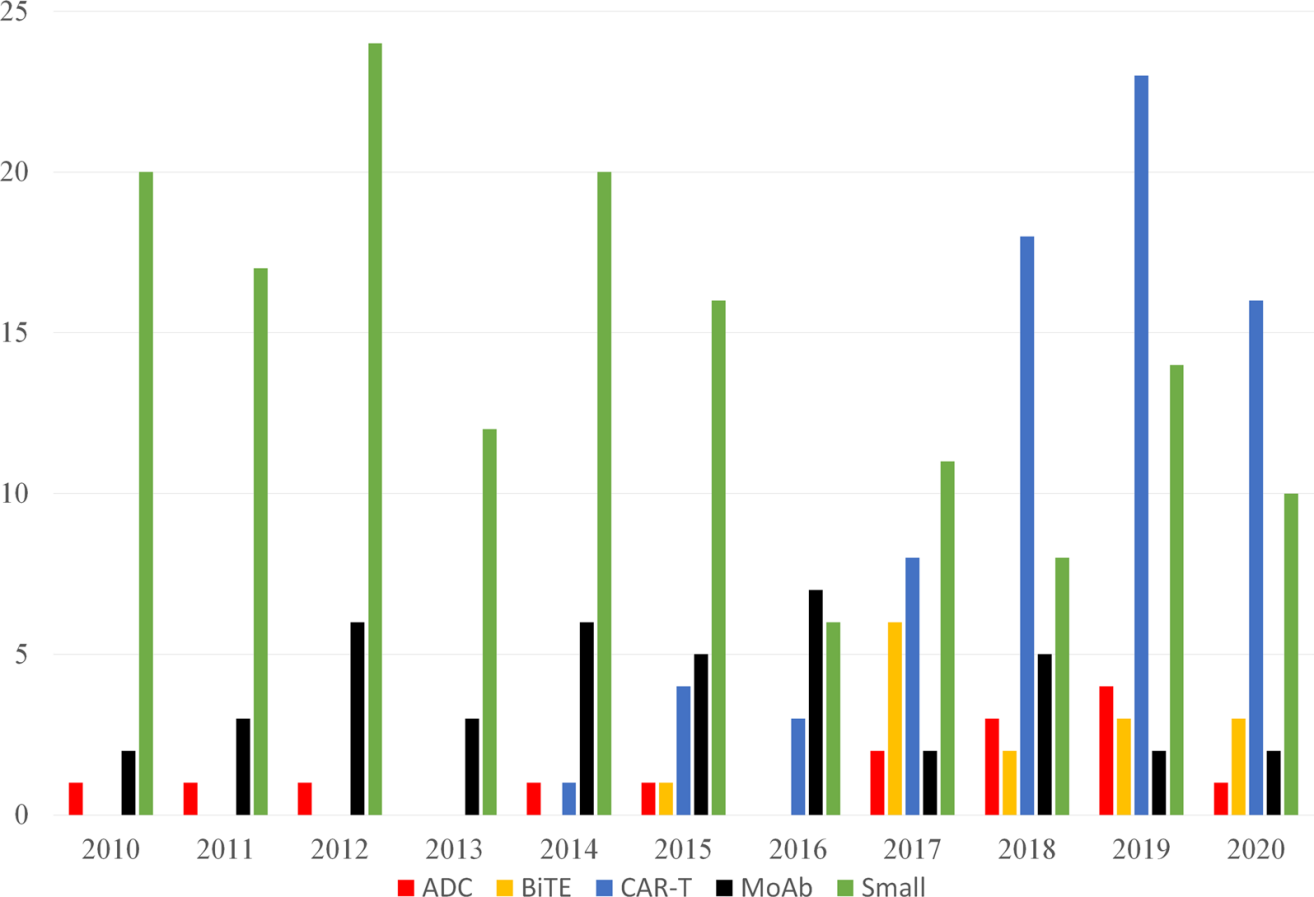
Phase 1 trials by year and type of studies, 2010–2020. *ADC* antibody–drug conjugate, *BiTE* bispecific antibody, *CAR-T* chimeric antigen receptor T cell, *MoAb* monoclonal antibody, *Small* small molecule inhibitor/modulator. Therapies not categorized as one of the above were excluded (59/363, 16%)

This review, as part of “First-in-human clinical trials for cancer therapy” thematic series, aims to discuss novel agents and emerging multiple myeloma therapies, with a special focus on data from early phase clinical trials presented at major 2019–2020 oncology meetings (Table 1) and actively recruiting registered Phase 1 clinical trials (Fig. 2). The number of substantially increased CAR-T trials since 2018 is especially noteworthy.

**Table 1:** Active clinical trials of novel therapeutic agents for RRMM presented at major oncology meetings, 2019–2020

Antigen	Product name* (NCT)	Study sponsor	Estimated enrollment	Country	Clinical Update reference
*CAR-T cells*					
BCMA	JNJ-68284528 (NCT03548207)	Janssen	118	United States, Japan	2020 ASH [7]
BCMA	bb21217 (NCT03274219)	Bluebird	74	United States	2020 ASH [8]
NY-ESO-1	GSK3377794 (NCT03168438)	GlaxoSmithKline	20	United States	2019 ASH [9]
BCMA /CD19	GC012F (NCT04236011)	Shanghai Changzheng Hospital	15	China	2020 ASH [10]
BCMA/CD38	BM 38CAR (ChiCTR1800018143)	Wuhan Tongji Medical College Union Hospital	20	China	2019 ASH [11]
BCMA	ALLO-715 (NCT04093596)	Allogene	90	United States	2020 ASH [12]
*Bispecific antibody (BiTE)*					
BCMA × CD3	Teclistamab JNJ-64007957 (NCT03145181)	Janssen	160	United States, the Netherlands, Spain, Sweden	2020 ASH [13]
BCMA × CD3	CC-93269 (NCT03486067)	Celgene	115	United States, Spain	2019 ASH [14]
BCMA × CD3	PF-06863135 (NCT03269136)	Pfizer	80	United States, Canada	2020 ASH [15]
BCMA × CD3	TNB-383B (NCT03933735)	Tenebio AbbVie	72	United States	2020 ASH [16]
BCMA × CD3	REGN5458 (NCT03761108)	Regeneron	74	United States, Belgium	2020 ASH [17]
GPRC5D × CD3	Talquetamab JNJ-64407564 (NCT03399799)	Janssen	245	United States, Netherlands, Spain	2020 ASH [18]
FcRH5 × CD3	BFCR4350A (NCT03275103)	Genentech	300	United States, Australia, Canada, Spain	2020 ASH [19]
*Antibody–drug conjugate (ADC)*					
BCMA, pyrrolobenzodiazepine	MEDI2228 (NCT03489525)	MedImmune	142	United States, Australia, Greece	2020 ASH [20]
BCMA, Shiga-like toxin-A subunit (engineered toxin bodies)	TAK-169 (NCT04017130)	Millennium Takeda	102	United States	2019 ASH [21]
*Monoclonal antibody (MoAb)*					
CD38	TAK-079 (NCT03439280)	Millennium Takeda	100	United States	2020 ASCO [22]
CD38 with interferon	TAK-573 (NCT03215030)	Millennium Takeda	151	United States	2020 ASH [23]
CD38	SAR442085 (NCT04000282)	Sanofi	78	United States, France, Spain	2020 AACR[24]
*Small molecule inhibitors and modulators*					
BCL-2 inhibitor	Venetoclax (NCT03314181, NCT01794520)	AbbVie	104, 117	United States, Australia, Belgium, Canada, Denmark, France, Germany, Norway	2019 ASH [25], 2019 ASH [26]
BCL-2 inhibitor	AT-101 (NCT02697344)	Mayo Clinic	10	United States	2019 ASH [27]
HDAC inhibitor	Alteminostat (NCT03150316)	Chong Kun Dang Pharamaceutical	18	South Korea	2019 ASH [28]
HDAC inhibitor	Chidamide (NCT04025450)	The First Affiliated Hospital of Soochow University	50 (high-risk NDMM)	China	2019 ASH [29]
Cereblon E3 ligase modulator	Iberdomide (CC-220) (NCT02773030)	Celgene	449	United States, France, Germany, Italy, Japan, the Netherlands, Spain, United Kingdom	2020 ASH [30]
Cereblon E3 ligase modulator	CC-92480 (NCT03374085)	Celgene	80	United States, Canada, Denmark, Finland, Spain, United Kingdom	2020 ASH [31]
MEK inhibitor	Cobimetinib (NCT03312530)	Hoffmann-La Roche	72	Czechia, Denmark, France, Germany, Netherlands, Norway, Poland, Spain, Sweden	2020 ASH [32]
MEK and BRAF inhibitors	Encorafenib and binimetinib (NCT02834364)	University of Heidelberg	12	Germany	2020 ASH [33]
*Other therapies*					
Peptidase enhanced cytotoxic	Melflufen (NCT03481556)	Oncopeptides	80	United States, Czechia, Spain	2020 ASH [34]
Peptidase enhanced cytotoxic	Melflufen (NCT02963493)	Oncopeptides	157	United States, France, Italy, Spain	2020 ASCO [35]

*ASCO* American Society of Clinical Oncology, *ASH* American Society of Hematology, *AACR* American Association for Cancer Research

*In cases where no unique product name was given, only the NCT number is listed to the bottom of the table (see Table 2 for example)

## Chimeric antigen receptor (CAR)-T cell

CAR-T therapy revolutionized immunotherapy in myeloma treatment since autologous stem cell transplant (ASCT) [36]. CAR-T therapy can be broadly grouped into three groups: single-target, multi-target, and universal CAR-T (Table 2). The ideal therapeutic CAR-T targets a cell surface antigen that is preferentially, and ideally exclusively, expressed on myeloma cells [37]. Resistance mechanisms such as “on target off tumor” recognition (expression of targeted antigens on normal cells) and “antigen escape” (loss of targeted antigens on tumor cells) pose ongoing therapeutic challenges in CAR-T therapy [38]. As a result, dual-target CAR-T strategies to increase precision of targeting have been proposed. Single-target CAR-T cells express one extracellular single-chain variable fragment recognizing tumor antigens, while dual-target CAR-T cells utilize co-stimulatory receptor design (separating the T-cell activation domain and the co-stimulatory domain into two separate CARs) or tandem CARs (two tandem-linked antigen recognition moieties coupled with one activation domain) [39]. Cytokine release syndrome (CRS) and neurotoxicity are significant adverse effects and important considerations for cellular-directed therapy (CAR-T and BiTE). These toxicities along with associated overall response rates for the treatments discussed are summarized in Table 3.

**Table 2:** Phase 1 and early phase 1 CAR-T trials for RRMM, as of December 31, 2020, with study start date after January 1, 2019

Antigen	Product name* (NCT)	Study sponsor	Estimated enrollment	Country
*Single-target CAR-T cells*				
BCMA	IM21 (NCT04537442)	Beijing Immunochina Medical Science and Technology	10	China
BCMA	CT053 (NCT03975907)	Carsgen	62	China
BCMA	NCT04271644, NCT04272151	Chongqing Precision Biotech	80 40	China
BCMA	C-CAR088 (NCT03815383)	First Affiliated Hospital with Nanjing Medical University	12	China
BCMA	NCT04626752	Hebei Senlang Biotechnology	50	China
BCMA	C-CAR088 (NCT03751293)	Hebei Yanda Ludaopei Hospital	10	China
BCMA	NCT04601935	Nanjing Legend Biotech	34	China
BCMA	C-CAR088 (NCT04295018)	Peking Union Medical College Hospital	10	China
BCMA	NCT04186052	PersonGen Bio (Suzhou)	10	China
BCMA	C-CAR088 (NCT04322292)	Tianjing Institute of Hematology and Blood Diseases	10	China
BCMA	NCT04637269	Xinqiao Hospital of Chongqing	16	China
BCMA	CARTBCMA ARI0002h (NCT04309981)	Institut d'Investigacions Biomèdiques August Pi i Sunyer	36	Spain
BCMA	NCT04155749	Arcelix	12	United States
BCMA	CT053 (NCT03915184)	Carsgen	70	United States, Canada
BCMA	Descartes-11 (NCT03994705)	Cartesian	18	United States
BCMA	CYAD-211 (NCT04613557)	Celyad Oncology	12	United States, Belgium
BCMA	CC-98633 (NCT04394650)	Juno/Celgene	80	United States
BCMA	PHE885 (NCT04318327)	Novartis	16	United States
CD4	NCT04162340	iCell Gene Therapeutics	12	China
CD22	NCT03999697	PersonGen BioTherapeutics (Suzhou)	10	China
CD44	MLM-CAR44.1 (NCT04097301)	MolMed S.p.A	58	Italy
CD138	NCT03672318	University of North Carolina	33	United States
GPRC5D	MCARH109 (NCT04555551)	Memorial Sloan Kettering Medical Center	36	United States
HA-1	MDG1021 (NCT04464889)	Medigene	29	The Netherlands
SLAMF7	NCT04499339	Wuerzburg University Hospital	38	Germany
SLAMF7	NCT03958656	National Cancer Institute	36	United States
TnMUC1	NCT04025216	Tmunity	112	United States
*Dual-target CAR-T cells*				
BCMA CD19	NCT04194931	The First Affiliated Hospital of Nanchang University	20	China
BCMA CD19	NCT04162353	iCell Gene Therapeutics	12	China
BCMA CD19	GC012F (NCT04236011)	Shanghai Changzheng Hospital	15	China
BCMA CD19	NCT04280328	Xian Xijing Hospital	18	China
BCMA CD19	NCT04603872	Zhejiang University	120	China
*Universal CAR-T cells*				
BCMA	ALLO-715 (NCT04093596)	Allogene	90	United States
BCMA	CTX120 (NCT04244656)	CRISPR Therapeutics	80	United States, Australia, Spain
BCMA	PBCAR269A (NCT04171843)	Precision BioSciences	48	United States
*Other*				
Anti-BCMA CAR-NK cells	NCT03940833	Asclepius Technology (Suzhou)	20	China
PD-L1 plus other antigen	NCT04191941	Timmune Biotech	9	China
CD34 + enriched hematopoietic progenitor cells expressing interferon-ɑ2	Temferon (NCT03875495)	Genenta	9	Italy
CD8 + T-cells expressing WT1, CD138, NY-ESO-1, and CS1	NEXI-002 (NCT04505813)	NexImmune	22	United States

^*^ In cases where no unique product name was given, only the NCT number is listed

**Table 3:** Overall response rates, cytokine release syndrome, and neurotoxicity associated with selected cellular-directed therapy

Antigen	Product name (NCT)	Study enrollment (n)	Median time to follow-up (months)	Overall response rate (%)	Cytokine release syndrome (%)	Neurotoxicity (%)
*CAR-T cells*						
BCMA	JNJ-68284528 (NCT03548207)	97	8.8	95	95	21
BCMA	bb21217 (NCT03274219)	46	8.5	55	67	22
BCMA /CD19	GC012F (NCT04236011)	16	7.3	94	88	0
BCMA/CD38	BM 38CAR (ChiCTR1800018143)	16	9.0	87.5	62.5	0
BCMA	ALLO-715 (NCT04093596)	19	2.0	60	24	0
*Bispecific antibody (BiTE)*						
BCMA × CD3	Teclistamab JNJ-64007957 (NCT03145181)	128	n/a	64	53	5
BCMA × CD3	CC-93269 (NCT03486067)	19	3.7	83.3	89.5	n/a
BCMA × CD3	PF-06863135 (NCT03269136)	18	n/a	33	61	n/a
BCMA × CD3	TNB-383B (NCT03933735)	38	n/a	37	21	13 (headache)
BCMA × CD3	REGN5458 (NCT03761108)	45	2.4	36	38	≥ 1
GPRC5D × CD3	Talquetamab JNJ-64407564 (NCT03399799)	137	n/a	78	47	5
FcRH5 × CD3	BFCR4350A (NCT03275103)	51	6.2	52	75	n/a

### Single-target CAR-T

Although there are numerous CAR-T candidates in clinical trials, only one CAR-T product is approved for clinical use in multiple myeloma. The most common cellular target in multiple myeloma CAR-T therapies currently is BCMA, a transmembrane glycoprotein in the tumor necrosis factor superfamily which is critical for B-cell differentiation to plasma cells and long-term plasma cell survival [40–42]. Furthermore, BCMA is preferentially expressed on plasma cells and not normal human tissue, including primary human CD34+ hematopoietic cells, making it an attractive target for CAR-T therapy [43]. The CAR-T candidate which has currently advanced the furthest in clinical development is idecabtagene vicleucel, a recent FDA-approved BCMA-directed CAR-T, that reported a 73% overall response rate (ORR) and minimal residual disease (MRD) negativity rate of 26% in relapsed refractory myeloma patients [6].

The LEGEND-2 trial (LCAR-B38M, NCT03090659, Phase 1, China) utilized a CAR-T design that incorporates two BCMA-targeting single-domain antibodies and one 4-1BB co-stimulatory domain It has enrolled 57 RRMM patients treated with at least 3 prior lines of therapy at four different sites with different conditioning regimens and number of CAR-T cells administered [44]. ORR was 88%, with CR achieved by 74% of evaluable patients (of which 93% achieved MRD-negativity). Median OS is not yet reached (at follow-up of 19 months), with OS at 18-months being 68%. Median PFS was 20 months for all patients, and 28 months for MRD-negative patients. Most common AEs were pyrexia (91%), CRS (90%, of which 82% were grade 1–2), leukopenia (30%), and thrombocytopenia (23%).

The CARTITUDE-1 trial (JNJ-4528, NCT03548207, Phase 1b, United States and Japan) has enrolled 97 RRMM patients treated with median 6 prior lines of therapy [7]. This study utilized the same CAR-T construct as in LEGEND-2 trial. Cyclophosphamide and fludarabine (Cy/Flu) was used as the conditioning regimen. ORR was 95% at median follow-up of 8.8 months, with 56% stringent complete responses (sCR) in evaluable patients. Median time to first response was 1.0 months, and median duration of response has not been reached. The 6-month PFS and overall survival (OS) rates were 87% and 94%, respectively. Most common AEs were CRS (95%, of which 96% were grade 1–2), neutropenia (91%), and anemia (81%). The rate of neurotoxicity was 21%, of which 90% were grade 1–2.

The CRB-402 trial (bb21217, NCT03274219, Phase 1, United States) has enrolled 46 RRMM patients treated with median 6 prior lines of therapy [8]. The study utilized a CAR-T construct based on idecabtagene vicleucel, consisting of one anti-BCMA single-chain variable fragment with one intracellular 4-1BB co-stimulatory domain [45]. Cy/Flu was used as the conditioning regimen. Clinical response was seen in 55% of evaluable patients with median follow-up time of 8.5 months. Median time to CR was 2.5 months and duration of response 11.9 months. AEs reported were CRS (67%, of which 94% were grade 1–2) and neurotoxicity (22%, of which 70% were grade 1–2). The CRB-401 trial (bb2121, NCT02658929, Phase 1, United States), which uses the same CAR-T construct as bb21217 but lacks the PI3K inhibitor bb007 added to bb21217 during ex vivo culture, similarly demonstrated efficacy and safety with median follow-up of 14.7 months and ORR of 76% in evaluable patients [46].

Other antigens most recently being explored for single-target CAR-T include CD4, CD22, CD44, CD138, HA-1, SLAMF7/CS1, TnMUC1 (Table 2), among others including CD19, CD38, NY-ESO-1, and numerous others described in literature [47]. The challenge to implement these differing antigen approaches is optimizing on-tumor targeting while minimizing toxic effects to normal tissue due to co-expression of myeloma markers. The NY-ESO-1 TCR T trial (GSK3377794, NCT03168438, Phase 1, United States) employs CAR-T targeted against NY-ESO-1, a cancer antigen expressed in diverse tumor types, including multiple myeloma [9, 48, 49]. Although only three patients were dosed with the trial medication at time of report, the authors report a 57% rate of positivity in bone marrow samples for NY-ESO-1 in eligible RRMM patients, suggesting NY-ESO-1 targeting may be a viable novel avenue of approach.

### Dual-target CAR-T

Several dual-target CAR-T trials have recently begun recruitment. All focus on BCMA and CD19 co-expressing CAR-Ts, which has demonstrated significant in vitro cytolytic activity on dual-expressor cells leading to complete tumor remission greater than that of BCMA or CD19 CAR-T alone [50].

The GC012F trial (GC012F, NCT04236011, Phase 1, China) enrolled 16 RRMM patients treated with median 5 prior lines of therapy [10]. The study used a dual-target CAR constructed by linking BCMA and CD19 single-chain variable fragments and Cy/Flu was used as the conditioning regimen. ORR was reported as 94% in evaluable patients, with 56% reaching MRD-negative sCR at median follow-up time of 7.3 months. CRS occurred in 88% of patients, of which 88% were grade 1–2. No neurotoxicity was observed.

The BM38 CAR trial (BM38 CAR, ChiCTR1800018143, Phase 1, China) enrolled 16 RRMM patients treated with at least 2 prior lines of therapy [11]. The study employed a dual-target CAR incorporating anti-CD38 and anti-BCMA antigen-recognition single-chain variable fragments linked in tandem to increase on-target sensitivity. Cy/Flu was used as the conditioning regimen. ORR was reported as 87.5% in evaluable patients, with 50% reaching sCR. Median PFS still has not been reached, but PFS at 9 months was 75%. The authors report CRS occurring in 62.5% of patients (60% had grade 1–2, while others had higher grades requiring tocilizumab). No dose limiting toxicities or neurotoxicity at levels higher than grade 3 were reported by the study authors at median follow-up of 36 weeks.

### Universal CAR-T/NK

All CAR-T therapies discussed thus far are autologous in origin, which pose advantages such as long-term persistence of engineered T cells due to the lack of an allogeneic reaction. However, autologous CAR-T therapy must be opportunely coordinated from harvesting to manufacturing and to final infusion. Some patients may not be able to afford the wait time of an additional 1–2 months due to rapid disease progression. Furthermore, previous lines of therapy that may negatively impact autologous T cells and T cell dysfunction are considerations following immunosuppression in the myeloma microenvironment [36, 51]. Thus, universal CAR-T/NK (UCAR-T/NK) therapy has been proposed as a treatment strategy to address these challenges, with advantages including batch-production and “off the shelf” availability and healthy T cells, but disadvantages including increased risk of graft-versus-host disease (GvHD) [52].

The feasibility of UCAR-T was described in an ex vivo platform which used bulk bone marrow biopsies from newly-diagnosed multiple myeloma (NDMM) and RRMM patients with high risk cytogenetics [53]. Following co-culture, young healthy donor anti-BCMA CAR-T cells against primary MM cells were able to achieve a specific anti-BCMA CAR-T cell killing rate of 13–73% while keeping target effects directed away from non-tumor cells. However, associated in vivo toxicity remains to be seen.

The ALLO-715 trial (ALLO-715, NCT04093596, Phase 1, United States) reported the first UCAR-T results, enrolling 19 RRMM patients treated with median 5 lines of prior therapy [12]. The study employed anti-BCMA UCAR-T cells with optimization to reduce the risk of GvHD and promote selective prolonged host lymphodepletion. Different conditioning regimens were used in treatment arms. ORR was reported as 60% in the arm with the highest efficacy, with 1 reaching sCR and all responders reached at least very good partial response (VGPR) and MRD-negativity. The most common AEs greater than grade 3 were anemia (41%), neutropenia (41%), and lymphopenia (29%). One grade 5 AE was reported as a sequela of multifocal pneumonia related to the conditioning regimen. No neurotoxicity or GvHD has yet been seen, with CRS reported in 24% of patients (75% grade 1, 25% grade 2).

Among ongoing UCAR-T trials, the CRISPR-Cas9 platform is being explored for the new generation of CAR-T cells, as the gene editing system is able to construct UCAR-T cells with defective T cell receptors and class I major histocompatibility complexes, avoiding GvHD and rapid rejection of the host immune system due to these modifications [54].

Furthermore, universal natural killer (NK) cell therapy is also being developed, although therapeutic challenges include specificity, persistence after infusion, and in vivo maximal activity of donor NK products. Phase 1 data of GDA-201 on 15 RRMM patients concurrently receiving elotuzumab showed no dose limiting toxicities and transient adverse effects without neurotoxicity, CRS, or GvHD. Response rate data has not been reported for myeloma patients, although the universal NK cell product demonstrated significant activity against multiple-refractory non-Hodgkin’s lymphoma with ORR of 73% [55].

## Bispecific T-cell engager antibodies (BiTEs)

Bispecific T-cell engager antibodies (BiTEs), the new kids on the block, comprise another important class of immunotherapies being explored in RRMM. Blinatumomab, an anti-CD19 BiTE for relapsed/refractory B-cell precursor acute lymphoblastic leukemia, was the first to be approved for clinical use in 2014 [56]. These constructs bind concomitantly to T cells and malignant cells, directing the cytotoxic effect of T cells selectively against tumor cells. Thus, BiTEs mimic CAR-T cells in mechanism and activity, although they do not require an extensive manufacturing process of viral transduction or donor T cell harvest. BiTEs join two single-chain variable fragments in tandem via a linker/connector [57]. An alternative DuoBody system is being developed, which utilizes a controlled antigen-binding fragment (Fab) arm exchange of IgG antibodies to create a new bispecific IgG [58]. Although several BiTE constructs are under investigation, none have yet been approved for clinical use for multiple myeloma. Numerous proposed trials from the last 2 years are currently in progress (Table 4).

**Table 4:** Phase 1 and early phase 1 bispecific antibodies (BiTE), antibody–drug conjugates (ADC), and monoclonal antibodies (MoAb) for RRMM, as of December 31, 2020, with study start date after January 1, 2019

Target antigens	Product name (NCT)	Study sponsor	Estimated enrollment	Country
*Bispecific antibodies (BiTE)*				
BCMA × CD3	REGN5459 (NCT04083534)	Regeneron	56	United States
BCMA × CD3	TNB-383B (NCT03933735)	Teneobio	72	United States
BCMA × CD3 × albumin (“tri-specific”)	HPN217 (NCT04184050)	Harpoon	70	United States
BCMA × CD16a	RO7297089 (NCT04434469)	Genentech	80	Australia, Denmark, Norway
CD28/38 × CD3	SAR442257 (NCT04401020)	Sanofi	57	United States
GPRC5D × CD3 and BCMA × CD3	Talquetamab and teclistamab (NCT04108195)	Janssen	100	United States, Canada, Germany, the Netherlands, Spain
*Antibody–drug conjugates (ADC)*				
BCMA, monomethyl auristatin-F [59]	GSK2857916 (belantamab mafodotin) (NCT03828292)	GlaxoSmithKline	14	Japan
BCMA, maytansinoid	CC-99712 (NCT04036461)	Celgene	120	United States, Canada
BCMA, Shiga-like toxin-A subunit (engineered toxin bodies) [21]	TAK-169 (NCT04017130)	Millennium Takeda	102	United States
CD46, monomethyl auristatin-F [60]	FOR46 (NCT03650491)	Fortis	50	United States
*Monoclonal antibodies (MoAb)*				
CD38	SAR442085 (NCT04000282)	Sanofi	78	United States, France, Spain
CD47	AO-176 (NCT04445701)	Arch Oncology	102	United States
IL-18	AEVI-007 (NCT04671251)	Aevi/Cerecor	30	United States

Teclistamab is a DuoBody bispecific BCMA and CD3 antibody which can be administered both intravenously and subcutaneously. The teclistamab trial (JNJ-64007957, NCT03145181, Phase 1, multiple countries) has enrolled 128 RRMM patients treated with median 6 prior lines of therapy in a dose-finding trial [13]. A response rate of 64% was achieved by all evaluable patients. Median time to first response was 1 month, and median duration of response was not reached, with responding patients remaining on therapy from 2 to 21 months. Most common AEs were anemia (55%), neutropenia (55%), and CRS (53%, all grade 1–2). Grade 3–4 AEs were reported by 39% of patients, with the most common being neutropenia (23%) and anemia (9%). Neurotoxicity was reported by 5% of patients (2% grade 3 or higher).

CC93269 is an asymmetric two arm IgG1-based human BiTE antibody that binds bivalently to BCMA and monovalently to CD3ε [61]. The CC-93269-MM-001 trial ( NCT03486067, Phase 1, multiple countries) has enrolled 19 RRMM patients treated with median 6 prior lines of therapy [14].ORR was 83.3% (≥ 6 mg CC-93269 in Cycle 1) in evaluable patients, with 33.3% achieving sCR and 75% achieving MRD-negativity at a follow-up interval ranging from 2.1 to 4.7 months. Grade 3–4 AEs were reported in 78.9% of patients, including neutropenia, anemia, infection, and thrombocytopenia, although no patients required dose modifications due to toxicity. CRS was reported in 89.5% of patients (of which 94% had grade 1–2). One death was reported due to CRS, with infection as a possible contributing factor.

The REGN5458 trial (NCT03761108, Phase 1, United States) has enrolled 45 RRMM patients treated with median 5 prior lines of therapy in a safety trial with REGN5458, a BiTE antibody also targeting BCMA and CD3 [17]. ORR was 36% across all dose levels, with 31% achieving at least CR. Most common AEs include CRS (38%, all grades 1–2), fatigue (18%), and nausea (18%), with dose-limiting toxicities including acute kidney injury and elevated liver transaminases. Serious treatment-associated AEs occurred in 22% of patients; the most common was CRS (11%).

The TNB383B trial (NCT03933735, Phase 1, United States) has enrolled 38 patients treated with median 7 prior lines of therapy [16]. TNB-383 is a BiTE targeting BCMA and CD3, with proprietary technology optimizing high affinity for BCMA with low-activating CD3 to minimize toxicity due to cytokine secretion from strong activation of CD3. ORR was 37% in evaluable patients, with a median duration of response of 9 weeks. The most common AEs were CRS (21%, all grade 1–2) and headache (13%); the most common grade 3–4 AEs were anemia (16%) and thrombocytopenia (13%).

The PF-06863135 trial (NCT03269136, Phase 1, United States and Canada) has enrolled 18 RRMM patients treated with median 7 prior lines of therapy in a dose-escalation safety trial [15]. PF-3135 is another BiTE antibody consisting of BCMA and CD3 targeting arms. ORR was 33% for evaluable patients (75% at the top two dosing tiers). Most common AEs were CRS (61%, all had grade 1–2), anemia (50%), and thrombocytopenia (39%). Grade 3–4 AEs were observed in 67% of patients; grade 4 AEs included lymphopenia (22%), thrombocytopenia (17%), and neutropenia (11%). Grade 5 AEs occurred in 17% of patients, but were not thought to be treatment-related.

Bispecific antibodies against other antigens on malignant cells aside from BCMA have also been created. The talquetamab trial (JNJ-64407564, NCT03399799, Phase 1, multiple countries) has enrolled 137 patients treated with median 6 prior lines of therapy in a dose escalation study with talquetamab, a BiTE antibody targeting GPRC5D and CD3 which can be administered intravenously or subcutaneously [18]. GPRC5D is an orphan receptor with high selective expression in primary myeloma cells [62]. ORR ranged from 78% (intravenous) to 67% (subcutaneous) in evaluable patients, with a median onset of response of 1 month. The most frequent AEs included anemia (50%), CRS (47%, mostly grade 1–2, with < 8% grade 3), and neutropenia (45%). Neurotoxicity was reported in 5% of patients (57% grade 1–2).

The GO39775 trial (BFCR4350A, NCT03275103, Phase 1, multiple countries) has enrolled 51 patients treated with median 6 prior lines of therapy in a safety trial evaluating BFCR4350A, a BiTE antibody targeting FcRH5 and CD3 [19]. FcRH5 is a membrane protein expressed on B and plasma cells, with almost 100% expression on observed myeloma cells [63]. ORR was 52% in evaluable patients, with 40% of these patients demonstrating durable response 6 months following therapy. The most common AEs were CRS (75%, of which 97% were grade 1–2), neutropenia (12%), and thrombocytopenia (10%).

## Antibody–drug conjugates (ADCs)

Antibody–drug conjugates consist of recombinant monoclonal antibodies covalently bound to cytotoxic chemicals (known as warheads or payload) via synthetic linkers [64, 65]. Since gemtuzumab ozogamicin for relapsed acute myelogenous leukemia became the first ADC to gain approval by the United States Food and Drug Administration (FDA) in 2010, there has been a total of eight additional approvals. Currently, three generations of ADCs were developed, with enhancing stability and potency while minimizing toxicity in each subsequent generation [64]. Belantamab mafodotin (GlaxoSmithKline) was the first agent approved for RRMM in August 2020 [5].

The MEDI-2228 trial (NCT03489525, Phase 1, multiple countries) has enrolled 82 RRMM patients treated with at least 3 prior lines of therapy in a dose-escalating study testing MEDI-2228, an ADC linking an anti-BCMA antibody which is conjugated to a DNA cross-linking pyrrolobenzodiazepine dimer [20]. Efficacy was seen at all dose levels, with ORR being 61% at the maximally tolerated dose. Most common AEs occurring at the maximal dose included photophobia (54%), thrombocytopenia (32%), and rash (29%), with no reports of keratopathy or visual acuity loss.

The TAK-169 trial (TAK-169, NCT04017130, Phase 1, United States) plans to enroll 114 RRMM patients in a two-part clinical trial to assess the safety and preliminary clinical activity of TAK169, a fusion protein comprised of an anti-CD38 antibody single chain variable fragment fused to a modified Shiga-like toxin-A subunit [21]. The proposed agent specifically binds to CD38+ cells, undergoes forced internalization of the target cell and retrograde transport to the cytosol, and finally irreversibly inactivates target cell ribosomes causing apoptosis. This mechanism is considered novel from standard ADCs and may be considered as a distinct class of therapeutics (engineered toxin bodies) with continued clinical success. In vitro, in vivo, and ex vivo assays were performed demonstrating significant direct tumor cell kill activity independent of the patient’s innate immune status. Current enrollment is ongoing (Table 4), and additional targets, including SLAMF7 with the same therapeutic construct, are being investigated [66, 67].

Other targeted antigens include CD123 (tagraxofusp, NCT02661022, phase 1, United States), an antigen expressed by plasmacytoid dendritic cells which is also found in the multiple myeloma bone marrow microenvironment [68]. Tagraxofusp is a CD123-directed cytotoxin fused to a truncated diphtheria toxin approved for blastic plasmacytoid dendritic-cell neoplasm [69]. Nine RRMM patients were treated with median 3 lines of prior therapy received tagraxofusp with pomalidomide and dexamethasone, with 56% reporting partial response in tumor reduction and > 50% decrease in peripheral blood plasmacytoid dendritic cell levels. The most common AEs were hypoalbuminemia (67%), chills (56%), and fatigue (56%); the most common grade 3–4 AEs were thrombocytopenia (44%) and neutropenia (33%), with no grade 5 events reported.

Finally, there are also efforts to create amanitin-based ADCs which are expected to have additional action against non-proliferating myeloma cells in contrast to ADCs utilizing microtubule- or DNA-targeting toxins which are more effective in actively dividing cells. HDP-101 is a new ADC targeting BCMA linked to amanitin especially aimed at targeting myeloma cells with *TP53* deletions in chromosome 17p [70]. Amanitin is active against RNA polymerase II, of which a major subunit is frequently co-deleted with *TP53* in cells with chromosome 17p deletions [71]. Preclinical studies demonstrated efficacy and tolerance [72]; clinical trials are forthcoming.

## Monoclonal antibodies (MoAbs)

Since 2015, monoclonal antibodies have become a stalwart of RRMM therapy, with recent approval of daratumumab as the frontline treatment in NDMM [73]. Currently, there are three FDA-approved monoclonal antibodies: daratumumab (anti-CD38), elotuzumab (anti-SLAMF7), and isatuximab (anti-CD38).

The TAK-079 trial (NCT03439280, Phase 1, United States) has enrolled 34 RRMM patients treated with median 4 prior lines of therapy in the TAK-079 trial [22]. TAK-079 is a subcutaneously administered anti-CD38 antibody that induces apoptosis via antibody-dependent cellular cytotoxicity and complement-dependent cytotoxicity [74]. At the recommended phase 2 dose, the authors report a preliminary efficacy of ORR 33% in evaluable subjects who received at least 6 cycles of therapy. The clinical benefit rate at the recommended phase 2 dose (minimal response or better) was 67%, with PFS not estimable given the current median follow-up of 7.5 months. The most common AEs were fatigue (21%), anemia (18%), neutropenia (18%), and leukopenia (15%), with only neutropenia being the only grade 3 AE. The only drug-related significant AE was grade 3 diverticulitis, with no grade 4 AEs, AEs leading to study discontinuation, or deaths secondary to AEs.

A related anti-CD38 therapeutic trial, TAK-573 (NCT03215030, Phase 1, United States) is currently in progress and has enrolled 59 patients with median 7 lines of prior therapy in a phase 1 dose-finding trial [23]. TAK-573, designed for directed interferon delivery contains an anti-CD38 monoclonal antibody fused to two attenuated interferon molecules. Response has been seen at nearly all dosing levels, with most common AEs being thrombocytopenia (83%, 47% grade 3 and above) and neutropenia (54%, 49% grade 3 and above).

SAR442085 is another anti-CD38 antibody currently beginning phase I trials (NCT04000282, Phase 1, multiple countries) [24]. The authors report that SAR442085 has a higher affinity for activating receptors on effector cells compared to daratumumab, resulting in an increased ability to engage CD16 with a higher level of NK cell activation.

Other target antigens currently being explored include CD47 (AO-176, NCT03834948, Phase 1, United States), an innate immune checkpoint found to be overexpressed on cancer cells [75–77]. AO-176 was found to not only promote phagocytosis of hematologic tumor cell lines but also directly target and kill tumor cells. Animal in vivo studies show complete tumor regression combined with bortezomib, including improved overall survival. Enrollment in phase 1 trial is ongoing for RRMM (NCT04445701).

## Small molecule inhibitors and modulators

**Fig. 3:**
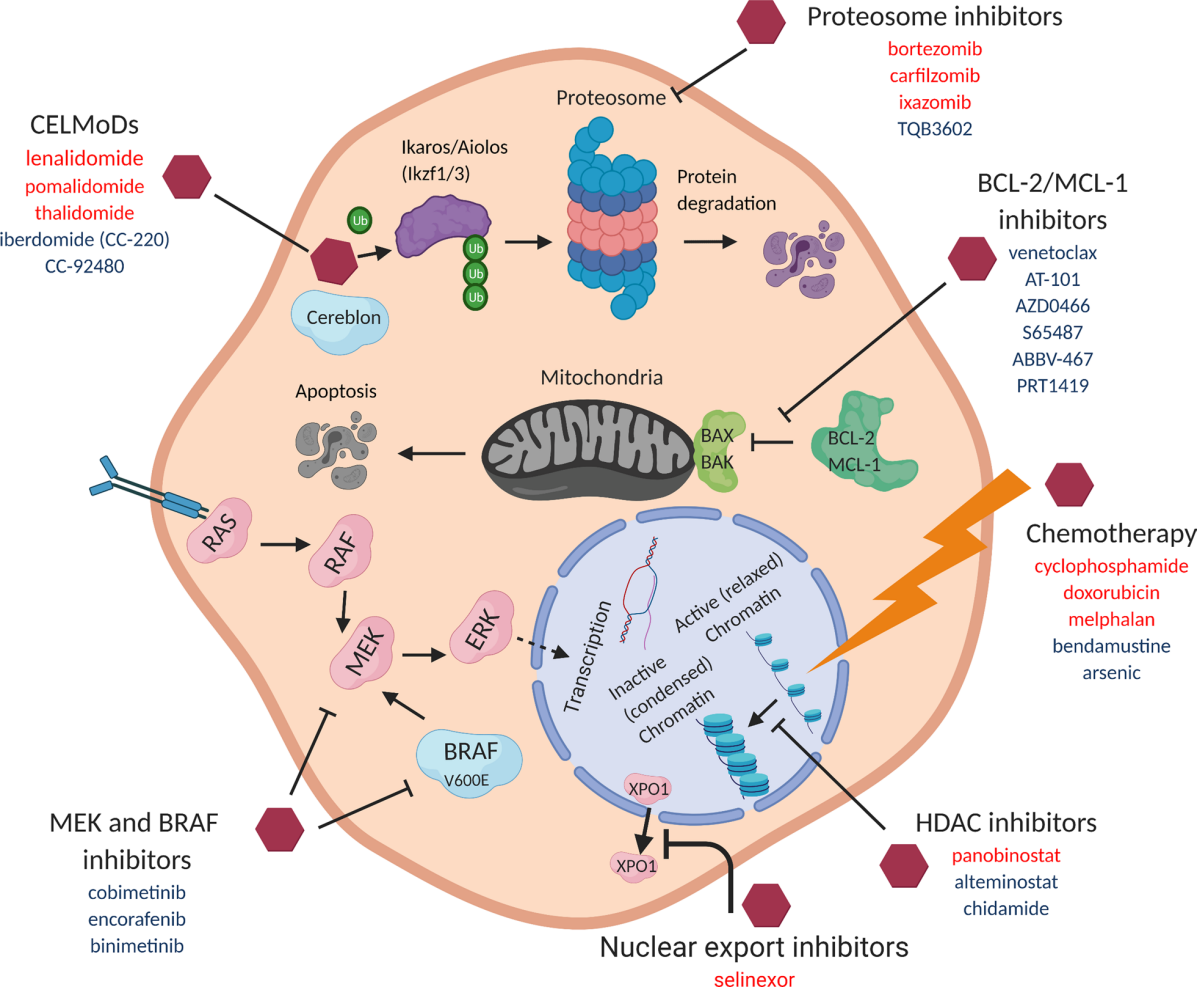
Mechanism of small molecule therapeutics in multiple myeloma therapy. FDA-approved medications are shown in red text and investigational therapies discussed in review text and Table 4 are shown in blue text

Small molecule inhibitors and modulators comprise the final major class of multiple myeloma therapy. Currently approved therapies include proteasome inhibitors (bortezomib, carfilzomib, ixazomib), immunomodulators (lenalidomide, pomalidomide, thalidomide), histone deacetylase inhibitors (panobinostat), and the novel nuclear export inhibitor, selinexor, which was approved July 2019 [78]. Figure 3 is a schematic of the major pathways of approved small molecule therapeutics described in this review, with current FDA-approved therapies for multiple myeloma highlighted in red text and investigational therapeutics in blue text. Phase 1 targeted therapy trials in this category initiated since 2019 are listed in Table 5.

**Table 5:** Phase 1 and early phase 1 small molecule inhibitors/modulators for RRMM, as of December 31, 2020, with study start date after January 1, 2019

Target pathway	Product name (NCT)	Study sponsor	Estimated enrollment	Country
Adenosine-2A receptor	Ciforadenant (CPI-444) (NCT04280328)	Corvus	28	United States
Arginase	INCB001158 (NCT03837509)	Incyte	98	United States, Germany, Spain
Bcl-2/Bcl-xL (apoptosis regulator)	AZD0466 (NCT04214093)	AstraZeneca	102	United States
Bcl-2	S65487 (NCT03755154)	Institut de Recherches Internationales Servier	60	Australia, France, Spain, United Kingdom
BTK (Bruton’s tyrosine kinase)	Ibrutinib (NCT03702725)	Alliance Foundation	28	United States
Cereblon E3 ubiquitin ligase (CELMoD – protein homeostasis)	CC-92480 (NCT03989414)	Celgene	215 (includes NDMM)	United States, Canada, Denmark, Finland, Spain, United Kingdom
IL-15 receptor (cytokine signaling)	NKTR-255 (NCT04136756)	Nektar	87	United States
IRF4 (lymphocyte transcriptional factor)	ION251 (NCT04398485)	Ionis	80	United States
JAK (Janus kinase/tyrosine kinase)	Ruxolitinib (NCT03773107)	Incyte	48	United States
LMP7 (proteasome complex subunit)	M3258 (NCT04075721)	EMD Serono	48	United States, France
Mcl-1 (apoptosis control)	ABBV-467 (NCT04178902)	AbbVie	54	United States, Australia, France, Israel, Japan, Spain, Taiwan
Mcl-1	PRT1419 (NCT04543305)	Prelude	36	United States
NEDD8 (ubiquitin-like protein)	Pevonedistat (NCT03770260)	National Cancer Institute	66	United States
p300/CBP (transcriptional coactivating proteins)	CCS1477 (NCT04068597)	CellCentric	90	United Kingdom
Proteasome inhibitor	TQB3602 (NCT04275583)	Chia Tai Tianqing	40	China
RAD51 (DNA repair protein)	CYT-0851 (NCT03997968)	Cyteir	165	United States
S100A9 (regulation of immune response and inflammation)	Tasquinimod (NCT04405167)	University of Pennsylvania	54	United States
TIGIT (T and NK cell immune receptor)	COM902 (NCT04354246)	Compugen	45	United States
TIGIT	Tiragolumab (NCT04045028)	Genentech	52	United States, Korea
Anti-TIGIT and anti-LAG	BMS-986016 and BMS-986207 (NCT04150965)	Multiple Myeloma Research Consortium	104	United States
TRAIL (tumor necrosis factor-related apoptosis-inducing ligand)	Eftozanermin alfa (ABBV-621) (NCT04570631)	AbbVie	40	United States, France, German, Italy, Japan, Spain
Specific targeted therapy based on mutations uncovered with genomic studies	MyDRUG (NCT03732703)	Multiple Myeloma Research Consortium	228	United States
Specific targeted therapy based on mutations uncovered with genomic studies	SMMART PRIME (NCT03878524)	Oregon Health and Science University	40	United States

### BCL-2/MCL-1 inhibitors

Venetoclax is an oral inhibitor of the BCL-2 protein which regulates the intrinsic mitochondrial apoptotic pathway as an anti-apoptotic. Venetoclax binds to BCL-2 and terminates the suppression of proapopotic proteins BAX and BAK, promoting cell death of the target cell (Fig. 3). Venetoclax is approved for chronic lymphocytic leukemia and acute myeloid leukemia and was further explored in RRMM, initially as single agent and later in combination with Velcade [79, 80]. Due to BCL-2 and cyclin D1 overexpression in *t*(11;14) multiple myeloma patients (up to 20%), venetoclax is increasingly being investigated as a therapeutic targeted agent in this patient population. Venetoclax trials in RRMM was halted due to findings from the Bellini trial with increased mortality seen in the venetoclax group, mostly due to an increased rate of infection [81].

Venetoclax was combined with Daratumumab in M15-654 trial (NCT03314181, Phase 1/2, multiple countries) which enrolled 48 RRMM patients in two groups [82]. In Part 1, patients with *t*(11;14) who received ≥ 1 prior line of therapy (PI and an IMIDs) were treated with VenDd. In Part 2, patients irrespective of *t*(11;14) status, non-refractory to PIs and failed 1–3 prior lines of therapy were treated with VenDVd. Frequent Grade ≥ 3 AEs in patients on VenDd were neutropenia (17%), hypertension (12%), fatigue and hyperglycemia (8% each), and in patients on VenDVd were insomnia (21%), diarrhea and thrombocytopenia (8% each). Eighteen patients had a serious AE (11 VenDd, 7 VenDVd), with pyrexia (n = 3) being most common. Median follow-up (VenDd/VenDVd) was 10 and 9 months. Overall response rate in VenDd/VenDVd was 96%/92% and 96%/79% had ≥ very good partial response rate. Median progression free survival and duration of response were not reached.

Novel Bcl-2 inhibitor AT-101 (NCT02697344, Phase 1, United States) enrolled 10 RRMM patients treated with median 2 prior lines of therapy in a safety and preliminary efficacy trial [27]. AT-101 is a pan BCL-2 inhibitor and the trial combined it with lenalidomide. ORR was 44% in evaluable patients, and median PFS was 8.1 months. Dose-limiting toxicities included grade 4 febrile neutropenia and thrombocytopenia. Most common grade 3–4 AEs included neutropenia, leukopenia, and thrombocytopenia. No patients experienced TLS.

Finally, MCL-1 is another antiapoptotic member of the BCL-2 family of proteins. Similar to BCL-2, MCL-1 binds to pro-apoptotic proteins BAK and BAX, and MCL-1 inhibitors remove that regulation and induce apoptosis via the intrinsic mitochondrial pathway [83]. Several MCL-1 inhibitor trials are underway, with promising preclinical data demonstrating in vitro tumor cell death and in vivo tumor growth inhibition [84, 85]. Thus far, phase 1 clinical trials are ongoing.

### Histone deacetylase (HDAC) inhibitors

The only HDAC inhibitor approved for multiple myeloma is panobinostat, approved in 2015 following the PANORAMA1 trial [86]. Panobinostat combined with bortezomib and dexamethasone achieved a median PFS of 12.0 months compared to 5.5 months in the placebo group. However, grade 3–4 AEs occurred in 96% of patients in the panobinostat group, compared to 82% in the placebo group. Panobinostat was ultimately approved with black box warnings for severe diarrhea (68% in the panobinostat cohort and 25% being grade 3–4, with 42% in the placebo group and 8% being grade 3–4) and EKG changes (prolonged QTc).

Since panobinostat, research into other promising agents in the family of HDAC inhibitors has continued (Fig. 3). There are 18 defined HDACs which play crucial roles in histone deacetylation, an important epigenetic modification that activates DNA transcription and is implicated in neoplastic growth. HDAC inhibitors reduce the activity of aberrant myeloma cells by inducing histone hyperacetylation, leading to cell cycle arrest and apoptosis [87].

The alteminostat trial (NCT03150316, Phase 1, South Korea) has enrolled 10 RRMM patients treated with median of 2.5 prior lines of therapy in a trial designed to assess alteminostat in combination with lenalidomide and dexamethasone [28]. Alteminostat is a novel pan-HDAC inhibitor with significant in vitro growth inhibition of multiple myeloma cell lines, especially in reducing the secretion of interleukin 6, which is associated with the proliferation of myeloma cells [88]. ORR was 70% in evaluable patients with 10% sCR, and median PFS was 7.7 months. Most common AEs included thrombocytopenia (80%), neutropenia (60%), and anemia (50%). No serious AEs, including cardiac disorders, were reported. The most common grade 3–4 AEs were neutropenia (70%), thrombocytopenia (60%), and anemia (60%).

The chidamide trial (NCT04025450, Phase 1, China) has enrolled 12 NDMM patients with high-risk characteristics in a clinical trial designed to assess chidamide in combination with bortezomib, lenalidomide, and dexamethasone [29]. Chidamide is a selective HDAC inhibitor targeting HDAC 1, 2, 3, and 10 which is currently approved in China for the treatment of peripheral T cell lymphomas [89]. Preclinical studies have indicated increased apoptosis and cell cycle arrest in treated myeloma cells [90]. In the clinical trial, ORR was 100% for evaluable patients, and 77% of patients obtained VGPR after one cycle. AEs included leukopenia, thrombocytopenia, and transaminase elevation. Grade 3–4 AEs were reported for thrombocytopenia and transaminase elevation. Two patients discontinued the study due to acute cardiac and acute renal failure.

In order to mitigate the toxicity associated with pan-HDAC inhibitors, preclinical studies have investigated selective HDAC6/8 inhibition (JBI-802, CVL608, CS3003) with demonstrable in vitro and in vivo myeloma growth inhibition [91–93]. These novel agents herald the arrival of more tailored approaches to HDAC inhibition in the future.

### Cereblon E3 ligase modulating drugs (CELMoD)

Lenalidomide, pomalidomide, and thalidomide are immunomodulators of cereblon E3 ligase, an essential enzyme in the cellular protein homeostasis machinery [94]. Proteins recruited cereblon E3 ligase are tagged with chains of ubiquitin and subsequently digested by the proteasome. CELMoDs bind to the surface of cereblon and recruit transcription factors such as Ikaros (IKZF1) and Aiolos (IKZF3) which are overexpressed in myeloma cells. These transcription factors are then ubiquitinated and targeted for subsequent destruction, leading to eventual host cell death (Fig. 3). There are currently several CELMoDs under clinical investigation [95].

The CC-220-MM-001 trial (iberdomide, NCT02773030, Phase 1/2, multiple countries) is a dose-escalation trial combining iberdomide with different treatment combinations. Thus far, it has demonstrated a favorable safety profile when combined with dexamethasone [96]. 19 patients received iberdomide in combination with daratumumab and dexamethasone (IberDd, median 4 prior lines of therapy) and 21 patients received iberdomide in combination with bortezomib and dexamethasone (IberVd, median 6 prior lines of therapy) [30]. ORR was 35% with IberDd and 50% with IberVd in evaluable patients. Median time to response was 4.1 and 4.9 weeks in both cohorts, respectively. Grade 3–4 AEs were reported in 78% with IberDd, most common being neutropenia (50%) and anemia (22%); 65% of patients with IberVd, most common being neutropenia (20%) and thrombocytopenia (20%).

The CC-92480 trial (NCT03374085, Phase 1, multiple countries) enrolled RRMM patients in a first-in-human study to evaluate the biological and clinical effects of CC-92480 and dexamethasone in heavily pretreated multiple myeloma patients [31]. The authors reported that Ikaros and Aiolos dose-associated degradation with concurrent T cell proliferation. CC-92480 led to rapid and sustained decreases in serum free light chains, although gaps in drug administration led to rapid rebound of these markers. Other CELMoDs (KPG-818, BTX-PHM) are currently under investigation, with favorable preclinical data [97, 98].

### MEK and BRAF inhibitors

Given the prevalence of MAPK pathway mutations in over 50% of newly-diagnosed myeloma patients [99], a trial investigating cobimetinib (cobimetinib, NCT03312530, Phase 1/2, multiple countries) enrolled 49 RRMM patients with median 4 prior lines of therapy in studies investigating the efficacy of MEK inhibition alone and combined with venetoclax (BCL-2 inhibition) and atezolizumab (PD-L1 inhibition) [32]. ORR was 0% for cobimetinib alone (C), 27% for cobimetinib combined with venetoclax (C + V), and 29% for cobimetinib combined with venetoclax and atezolizumab (C + V + A). Median duration of response was 11.5 months and 5.1 months for the C + V and C + V + A arms, respectively. The most common adverse effects (C/C + V/C + V + A) were diarrhea (33%/82%/91%), nausea (17%/50%/67%), and anemia (17%/46%/57%). Two cases of tumor lysis syndrome were reported, and treatment discontinuation were similar across the three arms at 14–18%.

Another trial using encorafenib and binimetinib (encorafenib and binimetinib, NCT02834364, Phase 2, Germany) enrolled 12 patients with median 5 prior lines of therapy in a study investigating BRAF and MEK inhibition in RRMM patients with BRAF V600E mutation; activating mutations of BRAF are found in up to 10% of RRMM patients[33, 100]. ORR was 82% with 27% of evaluable patients reaching CR. PFS assessment is still in progress, but duration of response exceeding 1 year was observed. Most common grade 3–4 AEs included anemia (25%), hypertension (25%), and thrombocytopenia (17%).

## Other investigational therapies

Other current investigational therapies include oncolytic targeted vaccines [101–104], enhancements to existing T cell-directed therapies [105], and other types of adoptive cell therapy aside from CAR-T cells [106, 107]. Trials studying chemotherapy agents such as bendamustine and arsenic have decreased in recent years in favor of targeted therapeutics [108, 109], although there are ongoing efforts to examine the efficacy of these agents in specialized populations [110–112].

Melphalan flufenamide (Melflufen), a peptidase enhanced cytotoxic derivative of melphalan with targeted delivery of melphalan via membrane bound aminopeptidase N (APN or AD13) [113], has demonstrated favorable in vitro and ex vivo effects on high risk multiple myeloma cell lines with del17p and TP53 mutations [114]. Melphalan flufenamide combined with dexamethasone was approved by the FDA in February 2021 based on HORIZON trial (NCT02963493) in RRMM patients who failed at least four prior lines of therapy [115].

## Conclusion

Immunotherapy has taken central stage thanks to relentless technological advancement and drug development. CAR-T/NK, BITEs, and ADCs, followed by novel small molecule targeted inhibitors/modulators, have shifted the multiple myeloma therapeutic paradigm. The ongoing trials with these novel agents offer patients various avenues of potential promising treatment options. Nevertheless, we are still facing many challenges ahead in RRMM management: how to achieve sustained deep remission, how to sequence and combine agents of different mechanisms of action, how to better manage and even prevent adverse effects, and how to improve and optimize convenience of drug administrations to patients.

Furthermore, drug resistance and immune escape are particularly complicated and challenging in the novel immunotherapies in RRMM, thus calling for more thought-provoking ideas to study mechanistic biology in basic and translational research [116, 117], better designed clinical trials to enrich patient populations stratified by meaningful biomarkers, and consolidated efforts to conduct high quality trials to generate practice changing data that can benefit our myeloma patients.

The financial costs associated with these novel therapies is another important issue for consideration: for instance, whether society, health system and patients can afford the expense of treatments such as CAR-T. However, patients can benefit from CAR-T as a promising therapy and enjoy a treatment break from chronic therapy [118, 119]. Treatment de-escalation in subset of the patients who achieved sustained deep response can not only spare patients from unnecessary long term drug toxicity, but also significantly reduce costs.

In summary, the advent of the novel myeloma therapeutic landscape will continue to evolve, paving the way to a new era of highly individualized treatment regimens and sophisticated disease burden assessment, moving the needle ever closer to an eventual cure.

## Data Availability

Not applicable.
